# Rising *Vibrio vulnificus* infections in Northern Europe linked to environmental reservoirs sustaining clinical pathogenicity

**DOI:** 10.1093/femsle/fnag028

**Published:** 2026-03-20

**Authors:** Yaovi Mahuton Gildas Hounmanou, Jules Beau-Gard Hougbenou, Jørgen Engberg, Hanne Marie Holt, Marianne Voldstedlund, Jens Andre Hammerl, Anders Dalsgaard

**Affiliations:** Department of Veterinary and Animal Sciences, Faculty of Health and Medical Sciences, University of Copenhagen, Stigbojlen 4, 1870 Frederiksberg, Denmark; Research Unit in Applied Microbiology and Pharmacology of Natural Substances, Laboratory Research in Applied Biology, Polytechnic School of Abomey-Calavi, University of Abomey-Calavi, Benin; Research Unit in Applied Microbiology and Pharmacology of Natural Substances, Laboratory Research in Applied Biology, Polytechnic School of Abomey-Calavi, University of Abomey-Calavi, Benin; Zealand University Hospital, Lykkebækvej 1, 4600, Køge, Denmark; Odense University Hospital, J. B. Winsløws Vej 4, 5000, Odense, Denmark; Statens Serum Institut, Artillerivej 5, 2300, Copenhagen, Denmark; DVG-Consultant Laboratory for Vibrio spp. in Food, Department Biological Safety, German Federal Institute for Risk Assessment, Max-Dohrn-Str. 8-1010589, Berlin, Germany; Department of Veterinary and Animal Sciences, Faculty of Health and Medical Sciences, University of Copenhagen, Stigbojlen 4, 1870 Frederiksberg, Denmark

**Keywords:** *Vibrio vulnificus*, vibriosis, climate-health, microbial genomics

## Abstract

*Vibrio vulnificus* is one of the most lethal marine pathogens, causing wound infections, septicemia, and necrotizing soft tissue infection with case fatality rates exceeding 20%–50%. In Northern Europe, incidence is rising alongside warming coastal waters. The genomic relatedness between environmental and clinical isolates and the long-term persistence of virulent clades in temperate waters remains poorly defined. An analysis of 117 *V. vulnificus* genomes, including 60 newly sequenced (36 environmental isolates and 24 clinical isolates from Denmark, 1994–2023), 27 publicly available Nordic clinical genomes, and 30 comparative reference public genomes were analysed. Comparative genomics, virulence gene profiling, pangenome analysis, and core-genome SNP (Single Nucleotide Polymorphism) phylogeny were used to define clade structure, persistence, and genetic determinants of pathogenicity. Four major clades were identified; three (C2–C4) persisted for up to three decades and contained both clinical and environmental isolates. Clonal pairs (≤40 SNPs) linked environmental and clinical sources across up to 8 years and multiple countries. The virulence gene repertoire including the major virulence factors MARTX toxin genes (*rtxA/B/C/D*), *chiRP* (chitin-regulated pilus), *flp*2/*tad*2 pili (fimbrial low-molecular-weight protein/tight adherence pili), *ilp*A (immunogenic lipoprotein A), and *ompU* (outer membrane porin), and *vvhA* (*V. vulnificus* hemolysin).) were present in both environmental and clinical isolates. All isolates carried the tetracycline resistance gene *tet*(34). We report that environmental aquatic reservoirs harbor fully virulent *V. vulnificus* lineages, making human infections exposure-driven rather than the result of emerging novel lineages.

## Teaser

Environmental reservoirs in the Baltic Sea sustain virulent *Vibrio vulnificus* lineages, making clinical infections primarily exposure-driven, with risk increasing due to climate-driven warming of coastal waters.

## Introduction

*Vibrio vulnificus* is a halophilic, Gram-negative bacterium capable of causing rapidly progressive wound infections, primary septicemia, and necrotizing soft tissue infection, with mortality exceeding 20%–50% in susceptible individuals (Archer et al. 2023). Clinical deterioration can occur within hours of symptom onset, necessitating urgent surgical intervention and aggressive antimicrobial therapy (Archer et al. 2023). Infections are most often acquired when open wounds are exposed to seawater or brackish water containing the pathogen, or during handling of contaminated seafood (Choi et al. 2021). Such exposures link an environmental reservoir directly to severe human disease, making *V. vulnificus* a model for studying how climate-sensitive aquatic bacteria translate environmental change into clinical risk.

In aquatic environments, the abundance of *V. vulnificus* is shaped by temperature, salinity, eutrophication, and nutrient availability factors increasingly altered by climate change (Amato et al. 2022, Ishida et al. 2023). Warmer surface waters, shifting salinity regimes, and phytoplankton blooms can enhance bacterial proliferation and persistence (Amato et al. 2022, Ishida et al. 2023). In Northern Europe, where historically cold waters limited *V. vulnificus* survival, rising summer sea surface temperatures have been accompanied by increased reports of human cases (Trinanes and Martinez-Urtaza 2021, Amato et al. 2022, Hounmanou et al. 2023a). In extreme heatwave years, such as 2018, the Baltic Sea recorded unprecedented human*Vibrio* case counts, including several fatalities (Trinanes and Martinez-Urtaza 2021, Amato et al. 2022, Hounmanou et al. 2023a). This expansion into new ecological niches underscores the need to understand the persistence and pathogenic potential of environmental lineages of *V. vulnificus*.

Phenotypic and molecular data have been used to classify *V. vulnificus* into three biotypes (BTs): BT1, predominating in human infections; BT2, causing disease in eels; and BT3, a rare biotype restricted to Israel that infects both fish and humans (Baker-Austin and Oliver 2018, Choi et al. 2021). Genomic studies have revealed substantial heterogeneity within the species. The *fpcrp* and *pilF* genes have been linked to fish- and human-pathogenic strains, respectively, often alongside the cytolysin/hemolysin gene *vvhA* (Lydon et al. 2021). Dimorphism in the *vcg* gene (*vcgE* in environmental isolates, *vcgC* in clinical isolates) and ribosomal RNA polymorphisms (*rrnA* in environmental strains, *rrnB* in clinical strains) have been proposed for strain differentiation (Lydon et al. 2021). However, these typing schemes often fail to account for the significant genomic overlap between clinical and environmental isolates (Lydon et al. 2021), limiting their value for risk prediction and molecular surveillance.

The pathogenicity of *V. vulnificus* depends on the presence and regulation of virulence genes, as well as their expression and host–environment interactions. However, it remains unclear whether environmental *V. vulnificus* strains are genomically distinct from clinical isolates in their repertoir of virulence genes. This is in contrast to the documented differences between environmental nontoxigenic *Vibrio cholerae* and clinical *V. cholerae* O1 lineages (Hounmanou et al. 2023b). First, without establishing whether environmental lineages can retain stable virulence determinants over decades, risk assessments may underestimate infection potential from long-standing reservoirs. Second, without high-resolution genomic data from environmental isolates, modeling how climate-driven environmental change affects disease incidence remains imprecise. Most surveillance in the Baltic and North Sea regions has been clinically focused, leaving the evolutionary dynamics and virulence maintenance of environmental strains poorly characterized.

We hypothesize that persistent environmental clades of *V. vulnificus* in Northern Europe retain clinically relevant virulence profiles and act as reservoirs for human infection. To address this, we sequenced and analysed 60 *V. vulnificus* isolates collected over nearly 30 years from Danish coastal waters and patients, including them in a study with 27 clinical isolates from other Nordic countries. Using comparative genomics, we resolved clade structure, assessed long-term persistence, and characterized virulence gene content, thereby linking environmental and clinical domains in a surveillance-relevant framework. These findings provide mechanistic insight into climate-sensitive pathogen persistence and inform targeted public health interventions.

## Materials and methods

### Study design and isolate collection

We analysed 117 *V. vulnificus* genomes, comprising 87 primary study genomes and 30 comparative reference genomes. Primary study genomes included 36 environmental isolates (32 newly collected and 4 archived isolates; Hounmanou et al. 2024), 24 Danish clinical isolates, and 27 publicly available Nordic clinical genomes from Sweden, Norway, and Finland (Amato et al. 2022).

Comparative reference genomes (*n* = 30) were included to study the strains in a global context.

**Figure 1:**
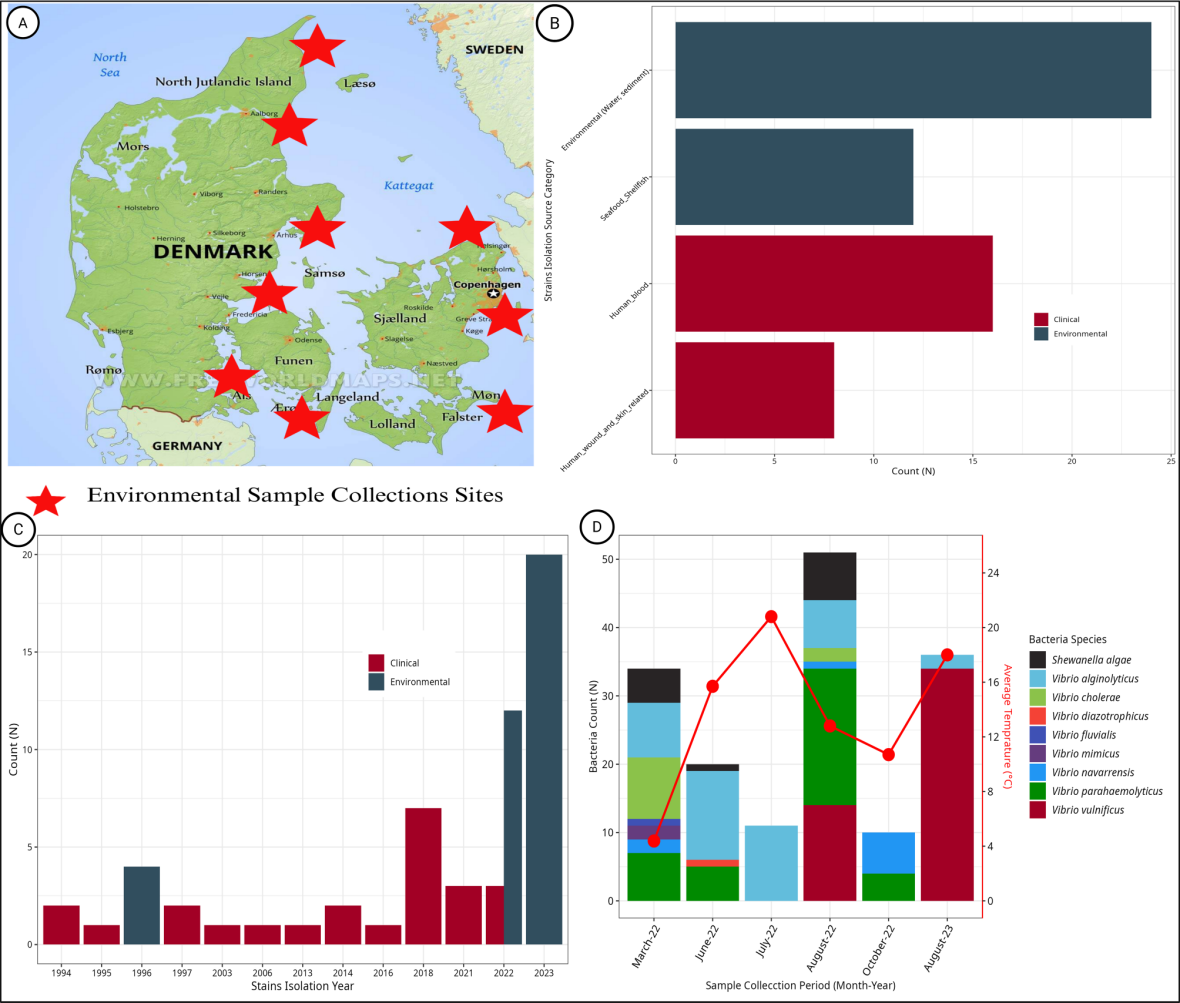
Sampling locations, bacterial diversity, and overview of clinical and environmental isolates analyzed in the present study. (A) Map of Denmark showing coastal sites (red stars) from the environmental survey conducted between March 2022 and August 2023. (B) Distribution of *V. vulnificus* isolates by source, including water, seafood, human blood, and wounds. (C) Temporal distribution of *V. vulnificus* isolates by source (clinical vs. environmental) from 1994 to 2023. Environmental samples have become more prevalent in recent years, while clinical samples are distributed across the timeline. (D) Monthly bacterial diversity in environmental samples collected in 2022–2023, showing species abundance, including *V. vulnificus*, over time. The red line represents mean sea surface temperature (°C) for each period.

The 32 environmental isolates obtained from the Danish Baltic coast between March 2022 and August 2023 (Fig. 1A) were obtained from seawater, blue mussels (*Mytilus edulis*), and green algae (*Ulva* spp.). Four archived environmental isolates from 1996, stored at the University of Copenhagen, were included to assess long-term clade persistence.

Clinical isolates (*n* = 24) were collected from hospital microbiology laboratories in Denmark between 1994 and 2022 from blood cultures, wound swabs, and skin biopsies of patients with laboratory-confirmed *V. vulnificus* infection (Fig. 1B). Finally, all 60 new strains collected in this study spanning 1994–2023 (Fig. 1C) were subcultured, and DNA was extracted for whole-genome sequencing (WGS) as previously described (Hounmanou et al. 2024).

Public Nordic genomes were retrieved from the NCBI database (BioProject IDs in Table S1) and represented primarily clinical isolates from wound and septicemia cases during summer seasons (Amato et al. 2022). Inclusion of these genomes enabled regional clade comparison and assessment of transnational strain connectivity.

### Isolation and species identification

Environmental samples were enriched in alkaline peptone water and plated on thiosulfate–citrate–bile salts–sucrose agar (Hounmanou et al. 2024). Colonies displaying *Vibrio*-compatible morphology were identified by MALDI-TOF mass spectrometry (Bruker Daltonics). Species identification for all isolates was confirmed by WGS and taxonomic assignment using GTDB-Tk v2.1.0.

### DNA extraction, sequencing, and assembly

Genomic DNA was extracted from overnight cultures using the Qiagen DNeasy Blood & Tissue Kit, following manufacturer protocols (Hounmanou et al. 2024). Sequencing libraries were prepared with the Nextera XT kit (Illumina) and sequenced on an Illumina MiSeq or NextSeq platform to produce 150–250 bp paired-end reads.

For public genomes, raw reads were downloaded when available; otherwise, assembled contigs were retrieved (Amato et al. 2022). Adapter and quality trimming was performed with Trimmomatic v0.39; assemblies were generated with SPAdes v3.15.4; and assembly quality was assessed with QUAST v5.2.0. Genome annotation was performed with Prokka v1.14.6 using a *V. vulnificus*-specific database.

### Multilocus sequence typing and pangenome analysis

Sequence types (STs) were assigned using mlst v2.22.0 with the *V. vulnificus* PubMLST scheme. The pangenome of the 87 study genomes was constructed using Panaroo v3.13.0 (clean mode, 95% protein identity threshold) to generate a presence/absence matrix and define core and accessory gene sets. Functional enrichment of accessory genes was tested using Scoary v1.6.16 with Benjamini–Hochberg correction (adjusted *P* < .05).

### Virulence and antimicrobial resistance gene profiling

Virulence-associated genes were identified using ABRicate v1.0.1 using the VFDB database (VFDB version of March 2024), and antimicrobial resistance (AMR) genes were detected with ABRicate v1.0.1 using the database option of ResFinder v4.1 (updated February 2024). All other parameters were kept as default. Virulence genes were grouped into functional categories capsule biosynthesis, cytotoxicity, iron acquisition, motility, quorum sensing, global regulation, and secretion/membrane interaction and summarized by source.

### *vcg* genotyping

Genotyping by the virulence-correlated gene (*vcg*) were determined by BLASTn alignment against reference *vcgE* and *vcgC* sequences retrived from UniProt (Hounmanou et al. 2024). Isolates were classified by allele profile (*vcgE* only, *vcgC* only, and *vcgE* + *vcgC*) and compared across sources and isolation years.

### Core-genome SNP phylogeny and clade definition

Core-genome SNPs were identified using Snippy v4.6.0 with *V. vulnificus* FORC_036 as the reference genome (Hounmanou et al. 2023b). Maximum-likelihood phylogenies were inferred with RAxML v8.2.12 using the GTR + GAMMA model with 1000 bootstrap replicates (Hounmanou et al. 2023b). Clades were defined as monophyletic groups supported by both phylogenetic topology and average nucleotide identity (ANI) clustering at ≥99.95% following existing thresholds (Wu et al. 2023). Clonal relationships were defined as ≤40 core SNPs, consistent with thresholds for recent common ancestry in bacterial genomic epidemiology and mostly because *V. vulnificus* are naturally heterogenous (Amato et al. 2022).

### ANI analysis and visualization

Pairwise ANI values were calculated with fastANI v1.3. ANI network clusters were visualized to assess genomic relatedness and the distribution of clinical versus environmental isolates.

#### Phylogenetic tree visualisation

Phylogenetic trees were visualized in iTOL v6, and pangenome data were displayed using Phandango. Spatial and temporal metadata were integrated and visualized using Microreact (Hounmanou et al. 2024). For tree visualization, iTOL was used for a circular visualization with annotations, utilizing metadata such as the presence of *vcg* markers, strain origins, and colored branches, along with clusters. Additional visualization was created using Microreact applying the tree out of iTOL. This tree was further annotated with clusters. Information about location and date of collection were added as metadata to provide a spatial and temporal distribution of the clusters.

## Results

### Seasonal occurrence and isolate diversity

Environmental detection of *V. vulnificus* in Denmark exhibited a seasonal pattern, directly linked to elevated summer sea surface temperatures. All the 32 environmental isolates collected between March 2022 and August 2023 were recovered in July or August, when mean sea surface temperatures exceeded 15°C (range, 15.1°C–22.7°C) (Fig. 1D). No isolates were obtained between October and May despite comparable sampling effort, underscoring a narrow ecological window for proliferation. Historical environmental isolates from 1996 were all collected in August during a warm-water period as well.

The 24 Danish clinical isolates spanned nearly three decades (1994–2022). The majority originated from wound infections (17/24; 70.8%), while five (20.8%) isolates were recovered from blood and two (8.3%) isolates from skin biopsies. Clinical cases were geographically distributed along the Zealand, Funen, and Jutland coastlines, which are regions characterized by intense summer recreational activities i.e. high exposure, thereby aligning epidemiologic risk with ecological expansion.

Across the 87 study genomes (36 contemporary environmental, 24 Danish clinical, and 27 Nordic public genomes), multilocus sequence typing (MLST) identified 29 STs. Most (21/29; 72.4%) were rare, represented by only one to three isolates, demonstrating substantial lineage heterogeneity. Several STs recurred in both clinical and environmental contexts across multiple years, strongly suggesting regional persistence and reemergence of pathogenic lineages under favorable conditions.

### Distribution of *vcg* genotypes

**Figure 2:**
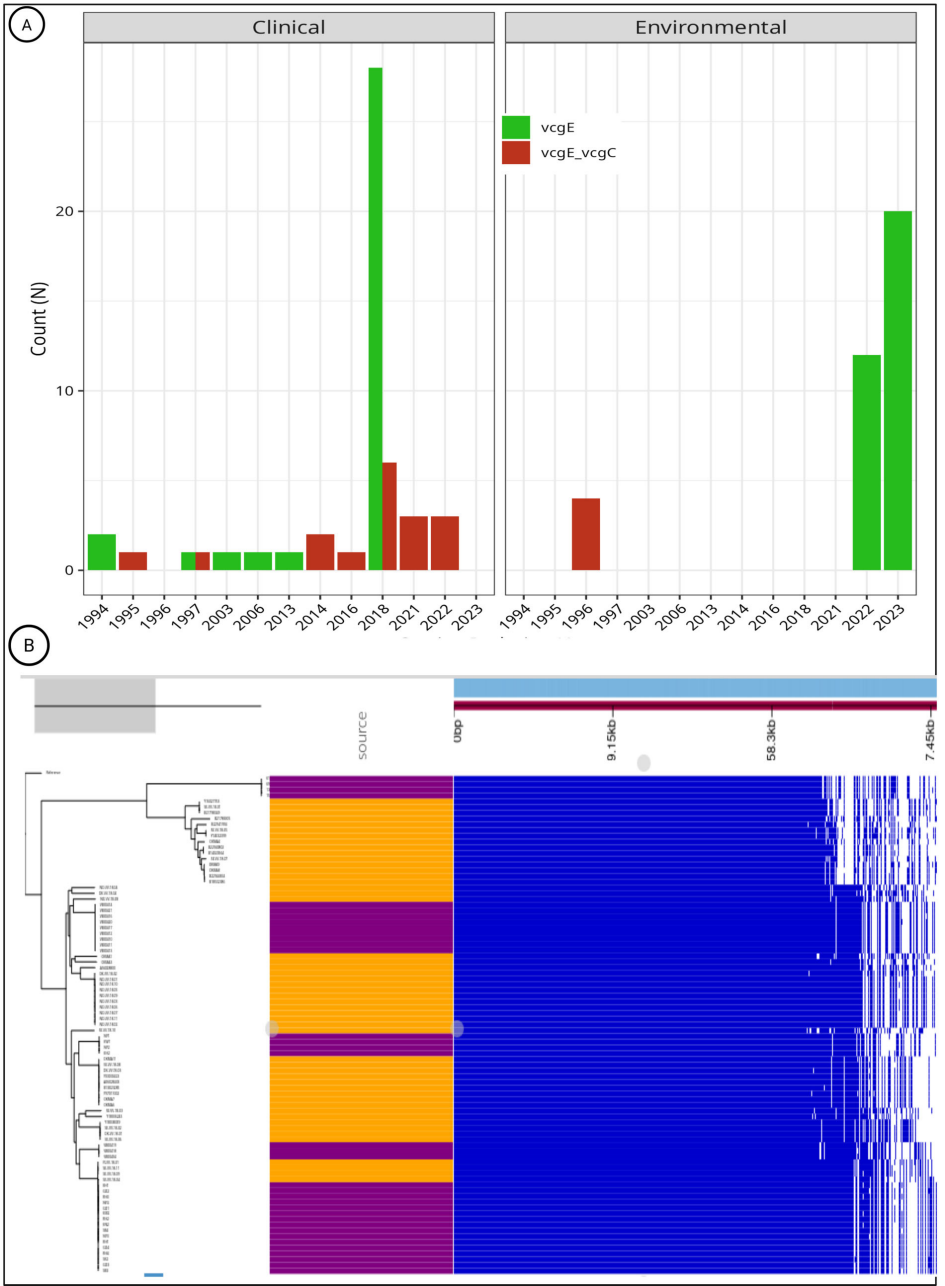
Genotypic characterization and pangenome comparison of *V. vulnificus* from clinical and environmental sources. (A) *vcg* genotyping of clinical and environmental isolates by year of isolation. Strains carry either the *vcgE* alone or the *vcgE–vcgC* combination. Notably, all clinical isolates after 2021 harbor the *vcgE–vcgC* pair. (B) Heatmap of pangenome analysis clustering *V. vulnificus* strains by gene content. No separation by isolation source is observed, with clinical and environmental strains interspersed in mixed clusters.

The virulence-correlated gene (*vcg*) locus revealed dynamic patterns. Overall, 52 of 87 isolates, from all clinical and environmental sources (59.8%) carried *vcgE* alone, while 35 (40.2%) harbored both *vcgE* and *vcgC* (Fig. 2A). *vcgC* alone was never detected. A temporal signal was evident among clinical isolates. Of the eight isolates collected after 2021, all (100%) carried both alleles, whereas only 3 of 16 (18.7%) earlier isolates (1994–2020) displayed dual-allele carriage. Several contemporaneous environmental isolates also carried both alleles, suggesting either horizontal acquisition of *vcgC* into *vcgE*-positive strains or expansion of preexisting dual-allele lineages. Importantly, identical allele combinations were found in clinical and environmental isolates recovered during the same years, supporting the absence of a genetic barrier at this locus between sources.

### Pangenome composition and clustering

The full pangenome of the 87 genomes is composed of 13 629 coding sequences, with 3389 (24.9%) forming the core genome (coding sequences present in at least 99% of the genomes) and 10 240 (75.1%) classified as accessory.

Analysis of the accessory genome showed no clustering by source, geography, or year (Fig. 2B). Instead, clinical and environmental isolates within the same clade often shared greater similarity with each other than with isolates of their own source type. Functional enrichment analyses revealed no significant differences in accessory gene content between sources (adjusted *P* > .05). These findings demonstrate that genomic variation is lineage-driven rather than source-restricted, further supporting the capacity of environmental aquatic strains to cause human infections.

### Virulence gene repertoire

**Figure 3:**
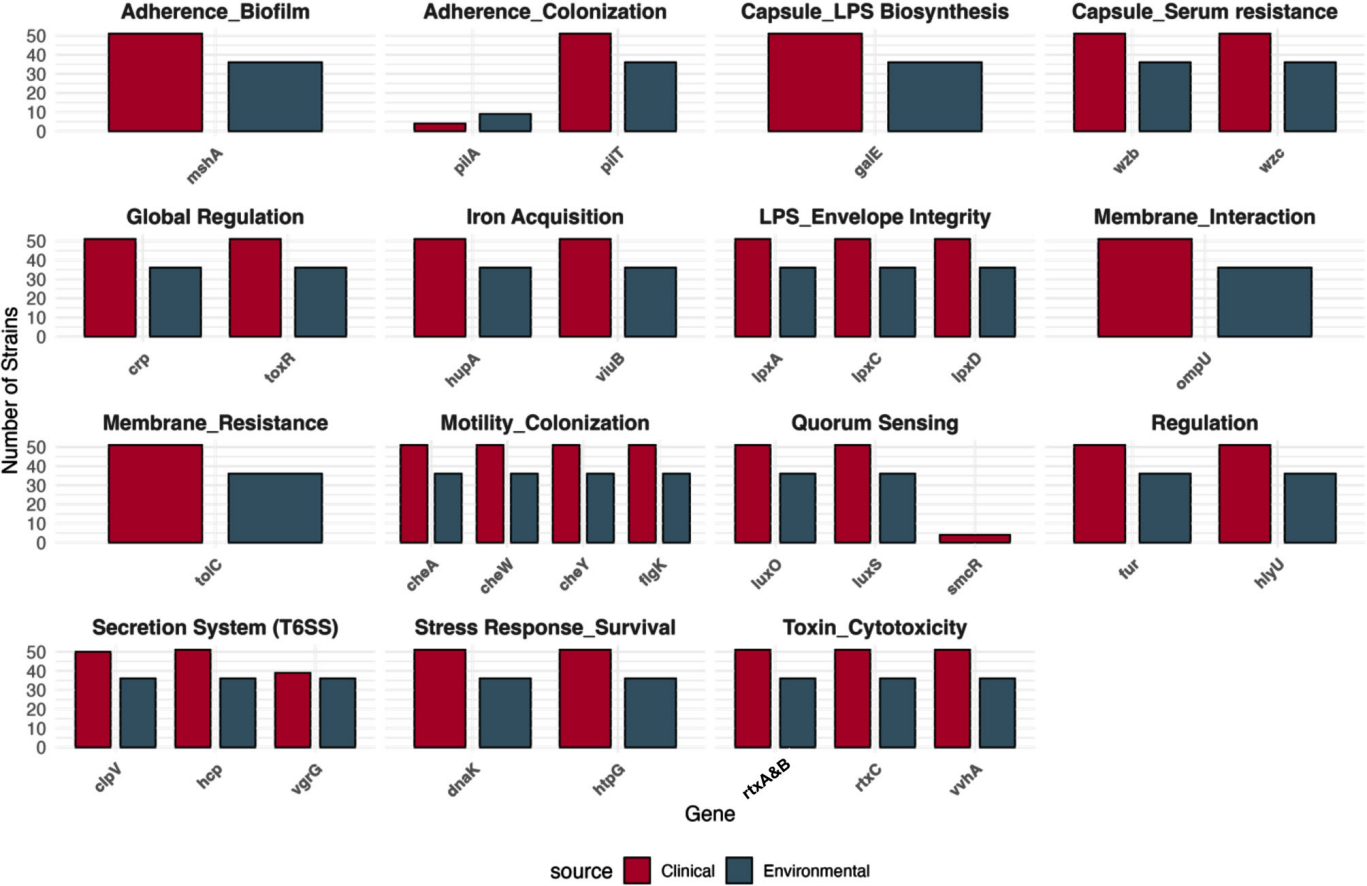
Strain-level distribution of virulence genes within functional categories, by source. Each bar represents the number of *V. vulnificus* strains from either clinical (red) or environmental (blue) origin that carry a given virulence gene, grouped by functional category. Genes on the *x*-axis are displayed per category (faceted panels), and bars indicate how many unique strains contained at least one copy of that gene.

Virulence profiling uncovered extensive functional overlap between clinical and environmental isolates (Fig. 3). Every isolate carried determinants spanning key pathogenic processes, such as (i) capsule formation (*wza* and *wzc*), essential for resistance to complement-mediated killing; (ii) cytotoxicity [MARTX genes (*rtxA, rtxB, rtxC*, and* rtxD*), and hemolysin *vvhA* gene], mediating host tissue destruction; (iii) iron acquisition (*hupA* and *vibB*), enabling survival in iron-limited host environments; (iv) motility and chemotaxis (*cheW* and *flgK*), facilitating host colonization and environmental dissemination; and (v) global regulation (*crp* and *toxR*), central to virulence gene expression. Other virulence factors, such as *chiRP* (chitin-regulated pilus), *flp*2/*tad*2 pili (fimbrial low-molecular-weight protein/tight adherence pili), *ilp*A (immunogenic lipoprotein A), and *ompU* (outer membrane porin) were detected in strains of both clinical and environmental sources.

The quorum-sensing regulator *smcR* was restricted to four clinical isolates (4.6%) distributed across distinct clades and years, suggesting sporadic, independent acquisition events. Apart from this rare gene, the virulence repertoire was >99% identical between environmental and clinical isolates. No major virulence factor was uniquely associated with clinical disease, and no determinant was absent from environmental isolates, underscoring the mechanistic equivalence in pathogenic potential across sources. Potential functional synergy with other virulence factors was not assessed.

### Antimicrobial resistance gene profile

All isolates carried *tet(34)*, a chromosomally encoded tetracycline resistance determinant along an insertion sequence IS5 element. No additional AMR genes were detected across decades, sources, or clades, highlighting remarkable stability of AMR within this population.

### Phylgenetic clade structure, diversity, and persistence

**Figure 4:**
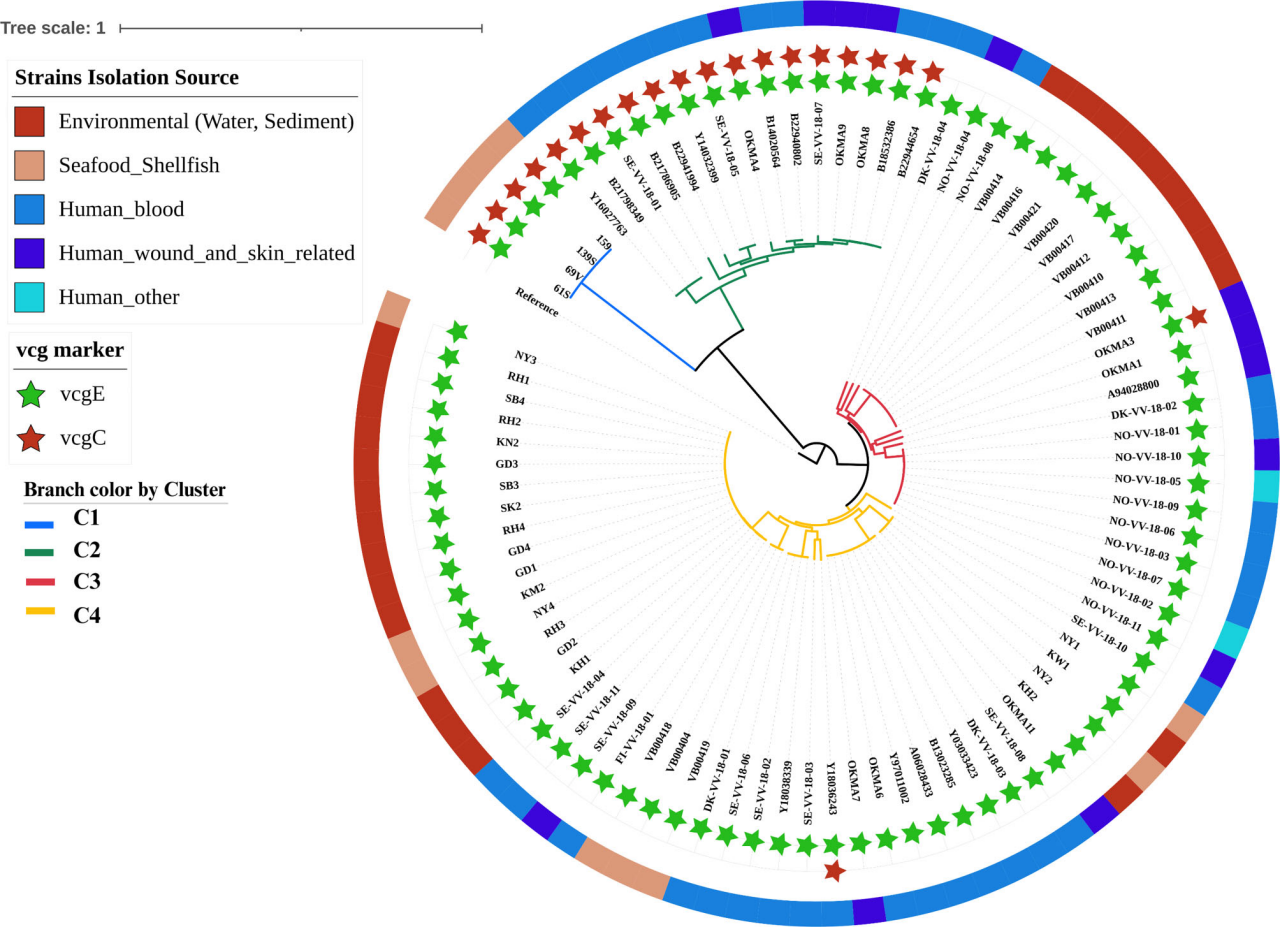
Phylogenomic clustering of clinical and environmental *V. vulnificus* showing the characteristics of the four key clades. Circular core-genome phylogeny annotated with *vcg* markers (*vcgE* and *vcgC*) and source categories. Isolates are grouped into four clades (C1–C4), with Clade C1 restricted to historical environmental isolates from 1996 and Clades C2–C4 containing mixed clinical and environmental strains.

**Figure 5:**
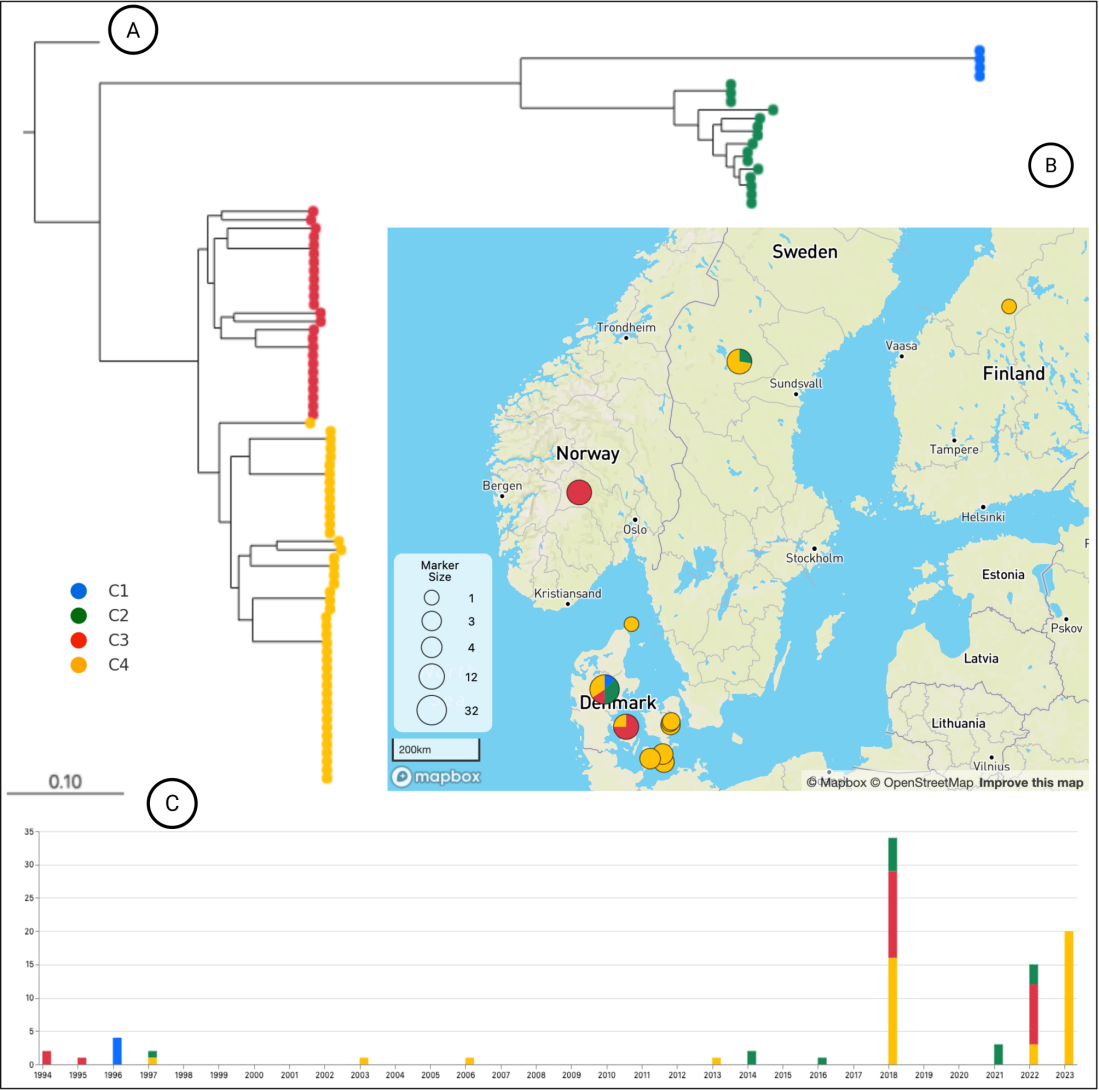
Phylodynamic and geographical clustering of *V. vulnificus* isolates. (A) Maximum-likelihood phylogenetic tree of the 87 *V. vulnificus* genomes, with branch colors indicating clade assignment (C1–C4). (B) Geographical distribution of *V. vulnificus* isolates across Denmark and the Nordic region, mapped by clade. Clades C2–C4 show widespread distribution, encompassing isolates from Denmark, Sweden, Norway, and Finland. Clade C1 is exclusively found in Denmark. (C) Temporal distribution of *V. vulnificus* isolates by clade and year of isolation (1994–2023). Clades C2–C4 show persistent detection across decades, with Clade C4 predominating after 2022. Clade C1, restricted to 1996, has not been detected since.

Core-genome SNP phylogeny coupled with ANI clustering (≥99.95%) resolved four major clades that we named C1–C4 encompasing clinical and environmental isolates collected over three decades (Figs 4 and 5A; Fig. S2).

A detailed analysis of each clade shows:

- Clade C1 (*n* = 4): Comprised exclusively of Danish environmental isolates from 1996, collected at a single site. Pairwise SNP distances ranged from 0 to 38, consistent with clonality and suggestive of a localized bloom event (Fig. 5; Fig. S1A).
- Clade C2 (*n* = 15): Included predominantly clinical (13/15; 86.7%), including isolates from Denmark and Sweden spanning 1997–2022. All carried both *vcgE* and *vcgC* (Fig. 4). Within C2, six isolates differed by ≤40 SNPs, while most others were separated by >100 SNPs, reflecting both clonality and long-term diversification (Fig. 5; Fig. S1A).
- Clade C3 (*n* = 25): Contained mixed-source isolates from Denmark and Norway spanning 1994–2022. A clonal subcluster of 2022 seawater and algae isolates differed by ≤40 SNPs, while Norwegian clinical isolates also formed a tight group (≤40 SNPs). In contrast, Danish clinical isolates within this clade displayed broad heterogeneity, with pairwise distances exceeding 2300 SNPs (Figs 4 and 5; Fig. S1B).
- Clade C4 (*n* = 43): Included the most diverse and geographically widespread clade, including isolates from Denmark, Norway, and Finland (1997–2023). Although most pairwise distances exceeded 100 SNPs, several clinical–environmental pairs clustered within ≤40 SNPs. Notably, Danish environmental isolates collected in 2022–2023 (SK2, SB4, SB3, RH4, RH3, RH2, and RH1) were ≤35 SNPs from the Swedish clinical isolate SE-VV-18–04 (2018), providing evidence of either long-term persistence or repeated introduction of near-identical clones (Figs 4 and 5A–C; Fig. S1C).

Across clades C2–C4, 12 clinical–environmental isolate pairs met the ≤40 SNP clonality threshold, spanning intervals of 1–8 years and originating from distinct countries. This constitutes direct genomic evidence that *V. vulnificus* strains established in aquatic environments can persist across multiple years and subsequently emerge as causes of human infection in geographically diversee settings.

Finally, the global phylogenetic comparison against reference genomes clustered all 87 isolates within the global clade previously known as biotype 1 (Fig. S1D).

## Discussion

This multidecade genomic investigation establishes that *V. vulnificus* strains inhabiting Northern European environmental aquatic reservoirs retain the full repertoire of virulence determinants and are genetically indistinguishable from patient-derived isolates in terms of virulence, demonstrating that the threat of severe human infection in temperate waters arises not from the de novo emergence of new pathogenic lineages but from recurrent exposure to persistent, established virulent clones. By combining archived isolates dating back nearly 30 years with contemporary clinical and environmental collections, we show that multiple clades of *V. vulnificus* (C2–C4) have persisted across decades and across national borders, with clonal matches between clinical and environmental isolates differing by fewer than 40 SNPs despite temporal separations of up to 8 years and recovery from distinct countries. These findings provide compelling genomic evidence for the ecological stability and long-term maintenance of specific pathogenic lineages in Baltic and North Sea waters and highlight that human infection risk is exposure-driven, shaped by seasonal oceanographic conditions and climate change, rather than by the recent evolution of novel hypervirulent variants (Leighton et al. 2023, Ma et al. 2023, Fernández-Juárez et al. 2024, Lee et al. 2024).

The persistence of clades across decades is particularly striking when compared with the population dynamics of other marine vibrios. Pandemic lineages of *V. parahaemolyticus* and epidemic *V. cholerae* O1 lineages are known to maintain long-term stability (Wu et al. 2022, Brumfield et al. 2023), but until now, evidence for similar ecological and genetic persistence of *V. vulnificus* in temperate waters has been limited. Our results demonstrate that *V. vulnificus* is not a transient colonizer of marine ecosystems but a stable component of the northern temperate microbial community, capable of persisting in multiple aquatic reservoirs, including estuarine sediments, seawater, and shellfish microbiota. The detection of clonal environmental subclusters such as the 2022 seawater and algae isolates in Clade C3 further supports the notion that local environmental niches provide stable habitats for long-term maintenance. In parallel, the presence of near-identical isolates across Denmark, Sweden, and Norway suggests regional connectivity, plausibly mediated by hydrodynamic circulation, shellfish trade, or migratory fauna, enabling clonal lineages to spread and reemerge across national borders. The post-2022 prominence of Clade C4 may reflect such ecological shifts, possibly influenced by marine warming, eutrophication, or other anthropogenic pressures (Riedinger et al. 2024).

Equally important is the observation that the virulence gene repertoire was indistinguishable between clinical and environmental isolates. Strains of both clinical and environmental sources carried major virulence genes, such as capsule biosynthesis genes (*wza* and *wzc*) that provide resistance to complement-mediated killing and phagocytosis, MARTX toxin genes (*rtxA, rtxB, rtxC, rtxD*, and *vvhA*) that drive cytotoxicity, tissue necrosis, and vascular injury, iron acquisition systems, such as vulnibactin biosynthesis (*hupA* and *vibB*) that are indispensable in iron-limited host environments, and global regulators (*toxR* and *crp*) that enable environmental sensing and coordinate virulence expression. The universal distribution of these genes across isolates confirms that environmental strains are fully equipped for opportunistic invasion of the human host and do not differ from the clinical isolates in terms of virulence factors, in line with previous studies documenting broad genomic heterogeneity but shared virulence potential in *V. vulnificus* (Phippen and Oliver 2015, D’Souza et al. 2018, Myintzaw et al. 2022, Leighton et al. 2023, Ma et al. 2023, Lee et al. 2024, Kim et al. 2014). This directly challenges the artificial dichotomy between “environmental” and “clinical” isolates and underscores that different aquatic environments constitute reservoirs of fully pathogenic *V. vulnificus*. Importantly, these observations are restricted to comparative genomic analyses of virulence gene repertoires and do not address differences in gene regulation or expression, which are known to modulate *V. vulnificus* pathogenicity and would require targeted transcriptomic or experimental investigation.

From a population genomics perspective, the absence of source-specific, geographic, or temporal segregation in accessory genome clustering, ANI networks, and core-genome phylogenetics demonstrates a high level of genetic connectivity across the Baltic Sea region. Clinical isolates were often more closely related to contemporaneous environmental isolates than to other clinical strains, further underscoring the ecological continuity of these lineages. This lack of geographic compartmentalization carries practical implications: localized monitoring of aquatic environments may underestimate the true diversity and distribution of *V. vulnificus*, as clonal lineages can appear in distant locations without intermediate detections. Similar genetic connectivity across space and time has been observed in other marine vibrios, including *V. cholerae* and *V. parahaemolyticus* (Wu et al. 2022, Brumfield et al. 2023), and emphasizes the need for cross-border genomic surveillance networks.

The seasonal pattern of recovery, restricted to months when sea surface temperature exceeded 15°C, aligns with laboratory-defined thermal thresholds for *V. vulnificus* growth (Hounmanou et al. 2023a, Fernández-Juárez et al. 2024, Riedinger et al. 2024). This observation is especially important in the context of climate change. The Baltic and North Seas have experienced unprecedented marine heatwaves over the last decade, with prolonged summer warming that extends the temporal window for *V. vulnificus* proliferation. In 2018, one of the warmest summers on record in Northern Europe, dozens of severe *Vibrio* wound infections, several fatal, were reported in Sweden, Finland, and Denmark (Wu et al. 2016, Amato et al. 2022, Hounmanou et al. 2023a). Our data provide the missing mechanistic context: virulent clades were already stably present in the environment, and their sudden epidemiologic emergence coincided with climate-driven increases in concentrations of *V. vulnificus* and human aquatic exposures, e.g. from recreational activities. This means that the risk of *V. vulnificus* infection is poised to rise in parallel with climate change, particularly affecting vulnerable populations, such as those with chronic liver disease, diabetes, or immunocompromised.

For clinicians, these findings reinforce the imperative to consider *V. vulnificus* in any rapidly progressive soft tissue infection or septicemia following marine exposure, even in temperate regions where the pathogen has historically been considered rare. Early recognition is essential, as delays in diagnosis are associated with mortality rates exceeding 30%, and prompt antimicrobial therapy combined with surgical intervention can be lifesaving. For public health, the genomic equivalence of clinical and environmental isolates means that monitoring environmental reservoirs provides a direct proxy for human infection risk. Surveillance systems that incorporate environmental sampling, real-time genomic typing, and integration with oceanographic data could enable predictive modeling of high-risk periods, facilitating targeted advisories such as warnings to avoid raw oyster consumption or marine exposure when sea surface temperatures exceed threshold values (Wu et al. 2016, Amato et al. 2022, Hounmanou et al. 2023a).

The One Health framework provides an ideal platform for integrating these approaches. Our findings highlight the value of combined environmental monitoring (temperature, salinity, plankton blooms, and shellfish microbiology), clinical microbiology (high-resolution genomic sequencing of wound and blood isolates), and real-time data sharing across national public health agencies.

A coordinated cross-border surveillance network in the Baltic–North Sea region would not only allow for early detection of emergent clones but also provide the foundation for predictive early warning systems that integrate climate data, microbial genomics, and human health outcomes. Extending this framework to other climate-sensitive marine pathogens could strengthen preparedness for a broad spectrum of emerging infectious threats.

Despite its strengths, this study is not without limitations. The isolate set, while spanning three decades, is constrained by the availability of viable archived strains and does not represent all clinical cases or environmental detections during the study period. Moreover, while genomic similarity establishes recent shared ancestry between clinical and environmental isolates, the study design cannot resolve the precise chains of transmission linking environmental reservoirs to human infection. Future work should combine genomic surveillance with case-control epidemiology, environmental metagenomics, and experimental infection models to quantify the risk posed by specific clades under defined environmental conditions and to map the pathways of transmission more precisely (Smalls et al. 2023, Jamal et al. 2024).

In conclusion, our findings provide genomic evidence that long-term environmental reservoirs of *V. vulnificus* in Northern Europe harbor strains with full clinical pathogenic potential, act as direct sources of human infection, and persist across decades without loss of virulence capacity. In the era of accelerating climate change and marine warming, infection risk is primarily a function of exposure opportunity rather than strain evolution. Proactive integration of environmental monitoring, genomic surveillance, and rapid public health communication will therefore be critical to mitigating the burden of *V. vulnificus* and other climate-sensitive marine pathogens in temperate coastal regions (Hounmanou et al. 2023a, Fernández-Juárez et al. 2024, Riedinger et al. 2024).

## Supplementary Material

fnag028_Supplemental_Files

## Data Availability

Reads submitted with project accession number PRJNA1240652 to Sequence Reads Archive. Figure S1 and Table S1 for metadata.
