# Supplementary material for: Rising Vibrio vulnificus infections in Northern Europe linked to environmental reservoirs sustaining clinical pathogenicity
Source: FEMS Microbiol Lett. 2026 Mar 20;373:fnag028. doi: 10.1093/femsle/fnag028 (PMC13070559; doi:10.1093/femsle/fnag028)
Supplement: fnag028_Supplemental_Files [file fnag028_supplemental_files.zip › Supplementary Figure.docx]

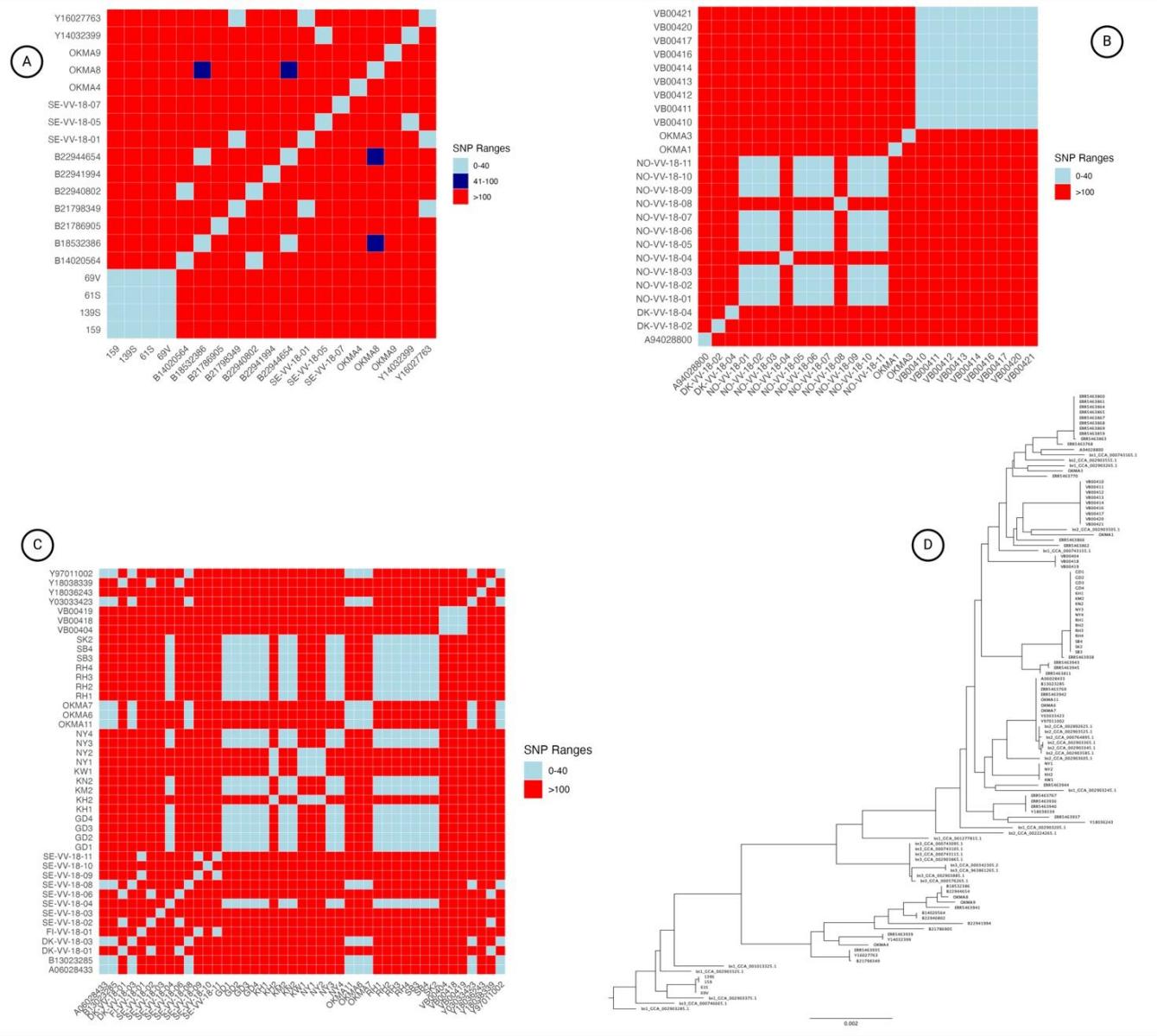


**Supplementary Figure Fig S1.** SNP-based clustering and phylogenetic relationships of *V. vulnificus* clades.

(A) SNP distance heatmap of Clades C1 and C2. Low SNP distances (in blue) within Clade C1 confirm its clonal nature, while Clade C2 shows moderate diversity, reflecting its mixed-source composition.
(B) SNP distance heatmap of Clade C3. Isolates within Clade C3 demonstrate moderate SNP diversity, indicating ongoing genetic divergence despite shared genomic characteristics across sources.
(C) SNP distance heatmap of Clade C4. Clade C4 exhibits significant SNP diversity, consistent with its predominance in recent years and its mixed-source nature, suggesting strong adaptive potential.
(D) Phylogenetic tree including 10 additional public genomes per biotype. The tree highlights the lack of phylogenetic grouping by biotype (BT1, BT2, BT3), underscoring the limitations of biotyping for resolving genomic relationships among *V. vulnificus* strains.


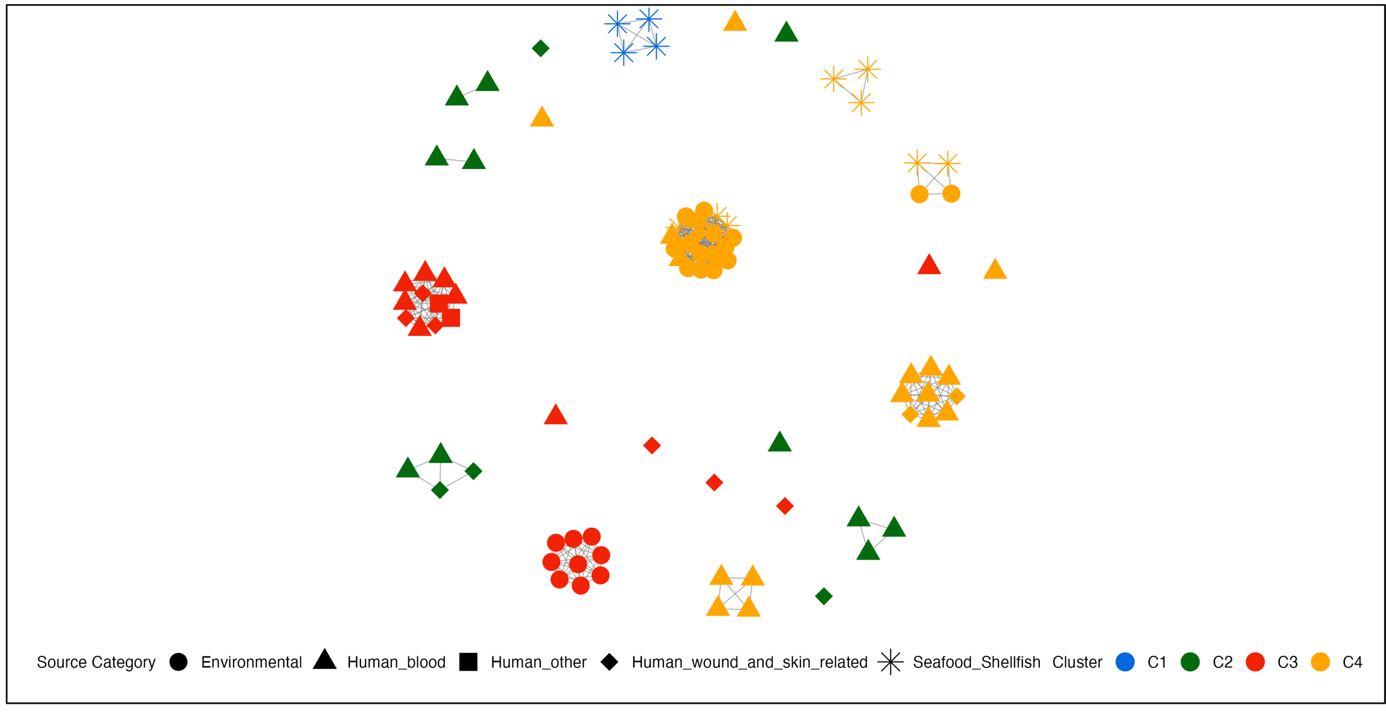


**Supplementary Figure Fig S2**: Average Nucleotide Identity (ANI)-based network of *V. vulnificus* isolates, clustered at a 99.95% similarity threshold. Nodes are colored by clade (C1–C4) and shaped by source category. The network demonstrates clonal relationships within Clade C1 and extensive genetic diversity across mixed-source Clades C2–C4.
